# The Effect of Dienogest on Deep Endometriosis Nodules Involving the Recto-Sigmoid Colon: A Prospective Longitudinal Long-Term Study

**DOI:** 10.3390/jcm14145164

**Published:** 2025-07-21

**Authors:** Carlos Andrés Contreras, Ignacio Brunel, Mónica Restrepo, Claudia Patricia Franco, María Clara Soto, José Carlos Vilches, Rodrigo Orozco, Stefano Guerriero, Juan Luis Alcázar

**Affiliations:** 1 Centro Colpodiagnóstico IPS, Medellín 005021, Colombia; cacongupi@gmail.com (C.A.C.); monirp@gmail.com (M.R.); claitofra@yahoo.com (C.P.F.); mariacsv2005@gmail.com (M.C.S.); 2 Department of Obstetrics and Gynecology, Hospital QuironSalud, 29004 Málaga, Spain; ibrunelg@gmail.com (I.B.); jcvilches@sego.es (J.C.V.); rodrigo.orozco@quironsalud.es (R.O.); 3 Department of Obstetrics and Gynecology, University of Cagliari, Policlinico Universitario Duilio Casula, 09042 Cagliari, Italy; gineca.sguerriero@tiscali.it

**Keywords:** endometriosis, rectum, sigmoid, ultrasound, symptoms, medical therapy

## Abstract

**Objective:** To assess the long-term effects of Dienogest on clinical complaints and nodule sizes in women affected by recto-sigmoid deep endometriosis (DE). **Methods:** This was a single-center longitudinal prospective observational study comprising a consecutive series of women affected by recto-sigmoid DE, who underwent medical treatment with Dienogest (2 mg daily continuous). All women underwent clinical visits and transvaginal sonography (TVS) with bowel preparation prior to starting therapy and at 3–6-month intervals for at least 12 months. Clinical complaints such as dysmenorrhea, dyspareunia and dyschezia were assessed using a visual analog scale (VAS). The DE recto-sigmoid lesion was measured in the three orthogonal planes. The lesion’s volume was estimated using the prolate ellipsoid formula. The maximum diameter and lesion volume were used for analysis. Patients’ complaints and lesion sizes before starting the treatment and at final follow-up were compared. **Results:** From January 2017 to July 2020, 125 patients were consecutively recruited (mean age: 37 years, ranging from 20 to 50 years). The median follow-up period was 47.8 months (range: 12–74 months). We did not observe a significant correlation between the severity of the symptoms and the lesion size prior to starting therapy. Clinical complaints improved significantly during treatment (88% of women were symptomatic at initial visit, versus 53% at final follow-up, *p* < 0.001). The median lesion volume significantly decreased (median initial volume vs. final volume: 1.1 mL vs. 0.9 mL, *p* = 0.017). However, the median maximum lesion diameter did not change significantly (26.0 mm vs. 25.0 mm, *p* = 0.779). **Conclusions:** Long-term Dienogest therapy significantly relieves clinical symptoms related to recto-sigmoid DE. This is accompanied by a significant reduction in the lesion volume but not the maximum lesion diameter.

## 1. Introduction

Endometriosis is a chronic and multifactorial gynecological disease characterized by the presence of functional endometrial tissue outside the uterine cavity [1]. Among its various presentations, deep pelvic endometriosis (DE) represents one of the most severe and symptomatic forms, defined as “endometrium-like tissue lesions in the abdomen, extending on or under the peritoneal surface”. They are usually nodular, able to invade adjacent structures, and associated with fibrosis and disruptions to normal anatomy [2]. This variant affects deep anatomical structures such as the uterosacral ligaments, the rectovaginal septum, the urinary bladder, the ureters, and, particularly frequently, the bowel, with the sigmoid rectum being the most commonly involved segment [3]. In fact, colorectal endometriosis affects 5% to 12% of patients with deep infiltrating endometriosis, with 90% of lesions located in the sigmoid colon or rectum [4,5,6,7].

Clinically, DE manifests with a variety of symptoms, including severe dysmenorrhea, deep dyspareunia, chronic pelvic pain, and, in many cases, infertility. This symptomatic complexity can lead to significant diagnostic delays, which worsens the impact on patients’ quality of life. Furthermore, the involvement of the gastrointestinal tract—particularly the rectum and sigmoid colon— may cause symptoms such as diarrhea, intestinal cramping, hematochezia, the passage of mucus, constipation and abdominal bloating, cyclic dyschezia, and tenesmus, which can mimic gastrointestinal diseases such as irritable bowel syndrome or inflammatory bowel disease, further complicating the initial clinical approach [8].

Treatment for deep pelvic endometriosis involving the bowel can be medical, surgical, or a combination of these [9]. Surgery seeks the complete removal of the lesions, particularly when structures such as the sigmoid colon are involved. The main surgical approaches currently used in the management of colorectal deposits include formal rectal resection and conservative surgery, either by shaving superficial endometrial deposits or by the full-thickness disc excision of deeper ones [10]. From a fertility perspective, bowel surgery has been shown to improve outcomes for both natural conception [11] and in vitro fertilization [12]. However, this type of intervention carries surgical risks and must be carefully considered [13,14]. On the other hand, recurrence may happen [15].

Medical management includes the use of hormonal therapies such as combined oral contraceptives, progestins, and GnRH agonists and antagonists, the primary goal of which is to suppress menstruation and relieve pain [8]. However, these strategies do not eradicate lesions, and their effectiveness in severe cases or in patients seeking fertility may be limited.

When patients complain about sub-occlusive symptoms, surgery should be considered as the first management option. However, when fertility is not an issue, medical therapy can be considered [8]. The hypothesis is that hormonal suppression could relieve symptoms related to DE and prevent the progression of the disease and the occurrence of new lesions. In addition, theoretically, medical therapy could have a potential benefit in preserving future fertility.

Several studies have evaluated its efficacy in terms of symptom control [8]. However, long-term studies analyzing the disease evolution of bowel DE using medical therapy are scant. Recent research using imaging tools such as magnetic resonance imaging and transvaginal ultrasound with bowel preparation has made it possible to monitor the progression or regression of lesions under hormonal treatment. Some of these studies suggest that certain regimens, such as progestogen monotherapy, can stabilize or even reduce the sizes of lesions without the need for immediate surgical intervention, although the evidence remains limited and such studies are often subject to strict inclusion criteria.

The aim of this study was to assess the long-term evolution of endometriotic rectal nodules in a series of women undergoing hormonal therapy with Dienogest as a progestin monotherapy.

## 2. Materials and Methods

### 2.1. Study Design

This was a retrospective analysis of prospective collected data from an observational cohort study performed at a single institution (Colpodiagnóstico Medical Center, Medellín, Colombia). This center specializes in diagnosing and managing pelvic endometriosis, performing 500–1000 examinations per month in women with suspected endometriosis. IRB approval (Pontifical Bolivarian University, Medellín, Colombia) was obtained, and all participating women signed an informed consent form after the nature of the study was fully explained. The STROBE guidelines were used for reporting in this study [16].

### 2.2. Study Population

Eligible patients were women diagnosed as having recto-sigmoid deep endometriosis who were candidates for non-surgical management. The recruitment period lasted from January 2017 to July 2020.

The single inclusion criterion was the diagnosis of at least one deep endometriosis nodule in the rectum or colon sigmoid. Exclusion criteria were as follows: pregnancy present at diagnosis or during follow-up, surgery for rectal or/and sigmoid colon during follow-up, study withdrawal, or loss to follow-up before 12 months after recruitment.

After inclusion, all women were offered medical hormonal therapy with Dienogest 2 mg as an oral pill daily.

### 2.3. Clinical Evaluation

At the first visit, the medical history and clinical complaints from each patient were obtained. A physical examination was also performed. For this study, the following data were collected: patient’s age and pain complaints, such as dysmenorrhea, dyspareunia, and dyschezia.

A visual analog scale was used to determine the pain intensity. A patient was considered as asymptomatic when the VAS for dysmenorrhea, dyspareunia, and dyschezia was scored as 0 or 1. A woman was considered as symptomatic whenever as the VAS score for dysmenorrhea, dyspareunia, and/or dyschezia was above 1. A VAS score of 2 to 4 was considered mild, a VAS score of 5 to 7 was moderate, and a VAS score of 8 to 10 was considered severe.

Physical examination findings during follow-up were not considered for this study.

### 2.4. Ultrasound Evaluation

All women underwent transvaginal ultrasound by several expert examiners according to the IDEA scanning protocol [17] using a Voluson S8, E6, or E8 machine equipped with 5–9 MHz endovaginal probes (GE Healthcare, Zipf, Austria). All women underwent bowel preparation prior to the ultrasound scan, consisting of Bisacodyl 5 mg the day before the examination and a sodium phosphate rectal enema (133 mL) two hours before the examination.

**Figure 1:**
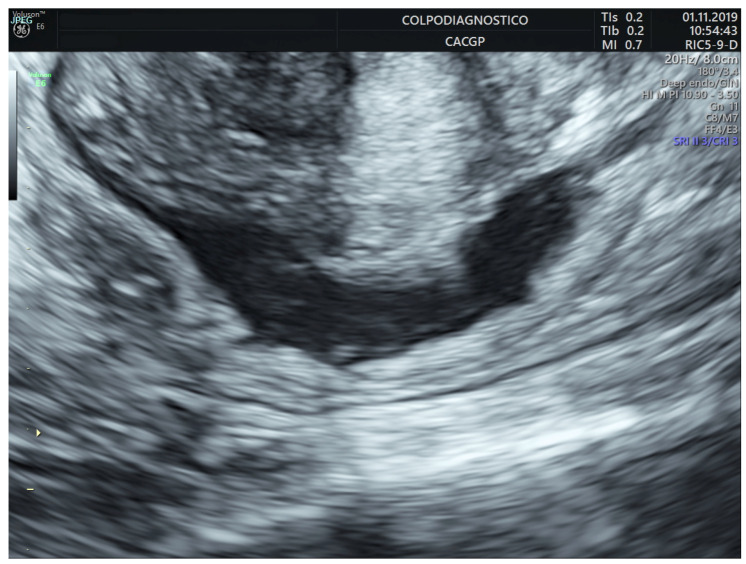
Transvaginal ultrasound image of an endometriotic nodule involving the upper rectum.

**Figure 2:**
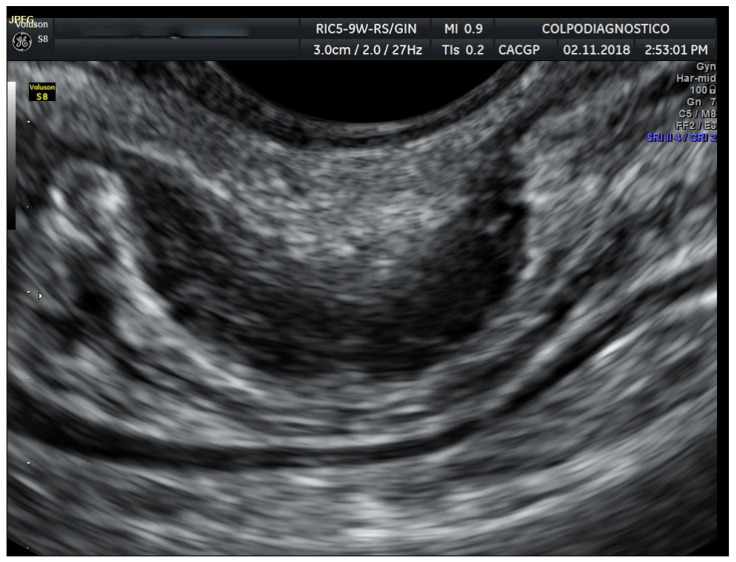
Transvaginal ultrasound image of an endometriotic nodule involving the sigmoid colon.

Rectal/sigmoid colon deep endometriosis nodules were observed as the thickening of the hypoechoic muscularis propria or as hypoechoic nodules, with or without hyperechoic foci with blurred, irregular, or spiculated margins (Figure 1 and Figure 2).

**Figure 3:**
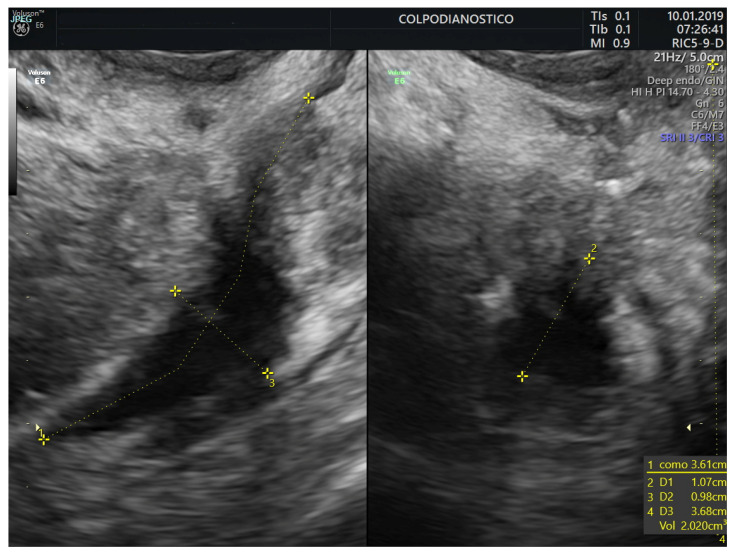
Transvaginal ultrasound image showing the measurements performed in an endometriotic nodule in the upper rectum.

**Figure 4:**
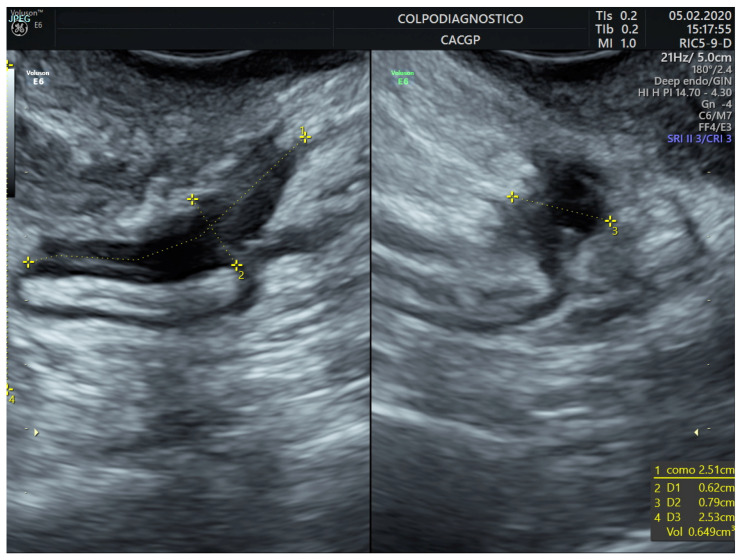
Transvaginal ultrasound image showing the measurements performed in an endometriotic nodule in the sigmoid colon.

**Figure 5:**
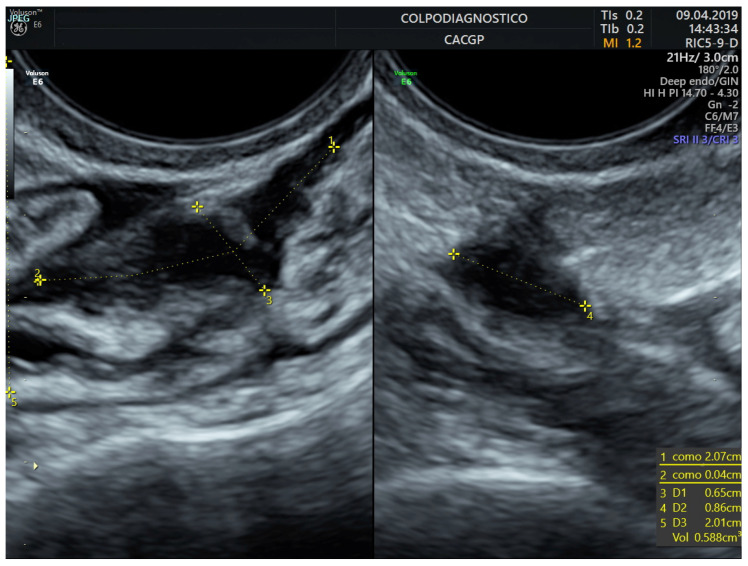
Transvaginal ultrasound image showing other measurements performed in an endometriotic nodule in the lower rectum.

The lesion was stated as single or multiple (multicentric and/or multifocal). All lesions were measured in all three orthogonal planes in mm (length, height, and width). The lesion volume was estimated using the ellipsoid prolate formula and expressed in milliliters (mL) (volume = length × height × width × 0.5233) (Figure 3, Figure 4 and Figure 5). Color or power Doppler was not used to assess lesion vascularization. For analysis, we used the lesion’s volume and the maximum diameter of the lesion (in almost 100% of cases, the largest diameter was the lesion’s length).

The identification of ovarian endometrioma, uterine adenomyosis, and deep endometriotic nodules in other locations, such as the bladder, ureter, rectovaginal septum, uterosacral ligaments, and parametrium, was recorded. Nevertheless, as stated above, for this study, we focused on rectal and sigmoid colon lesions.

### 2.5. Follow-Up

Patients were followed up with at 3, 6, and 12 months after recruitment during the first year and then at irregular intervals, depending on the ultrasound center’s agenda and the patient’s preference—some patients preferred twice a year and some preferred once a year, but visits took place at least once a year.

At each visit, data about pain complaints were recorded and a transvaginal ultrasound performed to assess lesion measurements, which were always performed using the same scanning protocol. Compliance with medical therapy was also confirmed and recorded.

For analytical purposes, we arbitrarily defined a lesion decrease as when the lesion’s maximum diameter or volume was 20% lower at the final visit as compared to the initial visit. Similarly, we defined lesion progression as when the lesion’s maximum diameter or volume was 20% higher at the final visit as compared to the initial visit. Finally, a lesion was considered stable when the variation in the maximum diameter or volume between the final visit and the initial visit was less than 20%.

### 2.6. Statistical Analysis

Categorical variables are presented as numbers and percentages. Continuous variables are presented as the mean with standard deviation (SD) or median with interquartile range (IQR), depending on the data distribution. The Kolmogorov–Smirnov test was used to assess the distribution of continuous data.

The chi-squared test or Fisher’s test was used to compare categorical variables, and Student’s *t*-test or the Mann–Whitney U-test for continuous variables.

To assess interobserver variability, two different examiners performed deep endometriosis nodule measurements in 15 patients. Both examiners performed measurements in the same patient a few minutes apart and blinded to each other. Interobserver variability was expressed as the difference between two measurement results obtained by the two different examiners.

Interobserver reproducibility was estimated according to the method of Bland and Altman, calculating the interclass correlation coefficient with 95% confidence intervals [18]. Differences between the measured values were plotted against the means of the two measurements to assess the relationship between the difference and the magnitude of the measurement [19]. Limits of agreement (mean difference ± 2 SD) were also calculated.

Sample size calculation was not performed.

The statistical analysis was performed using SPSS version 20.0 (SPSS Inc., Chicago, IL, USA) and the MedCalc software version 22.2009 (MedCalc, Mariakerke, Belgium). A *p*-value < 0.05 was considered statistically significant for all analyses.

## 3. Results

One hundred and forty-five women, presenting a total of 159 recto-sigmoid lesions, were recruited during the study period. Eleven women had two lesions, and one patient had three lesions. The last follow-up was in October 2023.

No woman became pregnant during the study period, and no woman underwent bowel surgery. However, 20 women (13.8%) had a follow-up period of less than 12 months and they were excluded. Therefore, a total of 125 women and 138 lesions were ultimately included in the analysis. For analytical purposes, in those cases with more than one lesion, the largest one was included in the analysis.

The patients’ mean age was 37 years old (SD: 5.9), ranging from 20 to 50 years old. Ten women (8%) had undergone a total hysterectomy prior to being included in the study. All women underwent Dienogest therapy as prescribed. Therapy compliance was above 95% in all women.

The median follow-up period was 47.8 months (interquartile range: 21.9, range: 12 to 74 months). Eight women underwent a hysterectomy during follow-up.

### 3.1. Clinical Changes During Follow-Up

At the initial visit, 110 women (88%) were symptomatic, mainly complaining of dysmenorrhea and/or dyspareunia (Table 1). The median VAS for dysmenorrhea was 8 (interquartile range: 10, range: 0 to 10), the median VAS for dyspareunia was 6 (interquartile range: 8, range: 0 to 10), and the median VAS for dyschezia was 5 (interquartile range: 8, range: 0 to 10).

Table 2 shows the distribution of symptom severity according to the VAS scores for dysmenorrhea, dyspareunia, and dyschezia at the initial visit.

At the final visit, 47% of women were asymptomatic. Table 3 shows the distribution of symptom severity at the final visit. At the final visit, the median VAS for dysmenorrhea was 0 (interquartile range: 10, range: 0 to 10), the median VAS for dyspareunia was 0 (interquartile range: 6, range: 0 to 10), and the median VAS for dyschezia was 0 (interquartile range: 10, range: 0 to 10). There was a significant reduction in the VAS scores for dysmenorrhea (*p* < 0.001), dyspareunia (*p* < 0.001), and dyschezia (*p* < 0.001). One hundred and eight out of 117 women who did not undergo a hysterectomy (92.3%) were amenorrheic at the final visit.

Table 4, Table 5 and Table 6 show the changes in the severity of dysmenorrhea, dyspareunia, and dyschezia from the initial to the final visit.

No patient presented sub-occlusive symptoms during follow-up, and, as stated above, no woman underwent bowel surgery.

### 3.2. Ultrasound Changes During Follow-Up

Regarding the interobserver variability analysis, both the maximum diameter measurement and lesion volume estimation were highly reproducible between observers. There were no statistical differences between observers for the lesion maximum diameter measurement and for lesion volume estimation (Table 7). The mean difference for the maximum diameter measurement was 0.8 mm (SD: 1.8) and that for lesion volume estimation was 0.33 (SD: 0.69).

**Figure 6:**
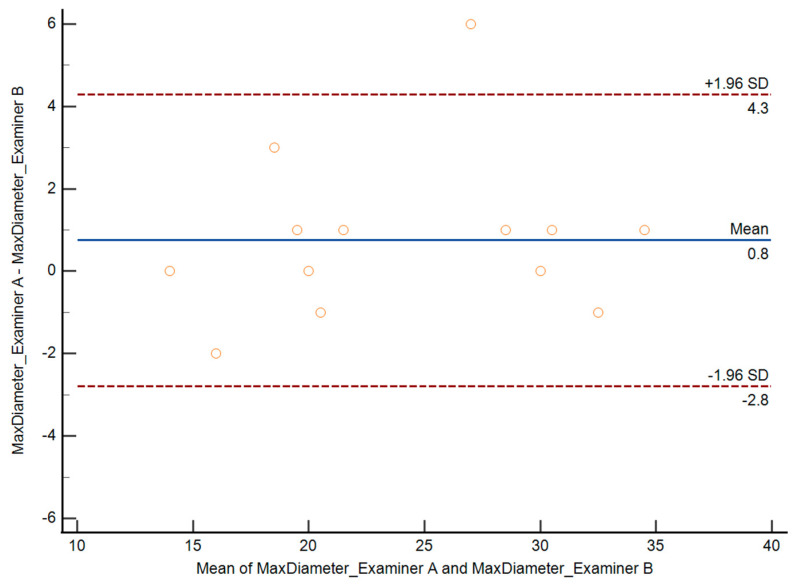
Bland–Altman plot showing the difference in the maximum diameter of the lesion for both examiners against the mean (blue line, SD shown as dotted red line).

**Figure 7:**
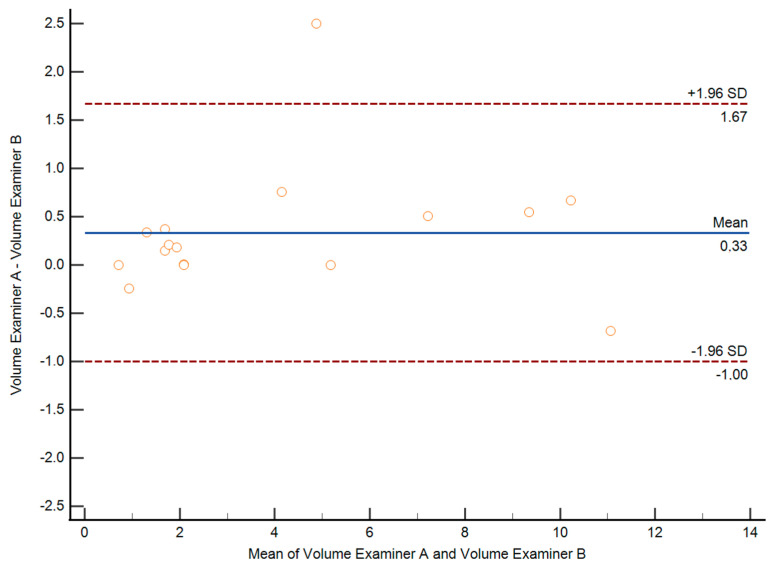
Bland–Altman plot showing the difference in the lesion volume for both examiners against the mean (blue line, SD shown as dotted red line).

The intraclass correlation coefficient for the maximum diameter measurement was 0.980 (95% CI: 0.942–0.993). The intraclass correlation coefficient for lesion volume estimation was 0.950 (95% CI: 0.857–0983). Graphics showing the relationships between the differences and magnitudes of the measurements are depicted in Figure 6 and Figure 7.

At the initial visit, the lesion’s median maximum diameter was 26 mm (interquartile range: 16, range: 7 to 89 mm), whereas the median lesion volume was 1.1 mL (interquartile range: 1.5, range: 0.1 to 13.4 mL). We did not observe a significant correlation between the severity of the symptoms and the lesion size prior to starting therapy (Table 8, Table 9 and Table 10). This finding remained during follow-up.

**Figure 8:**
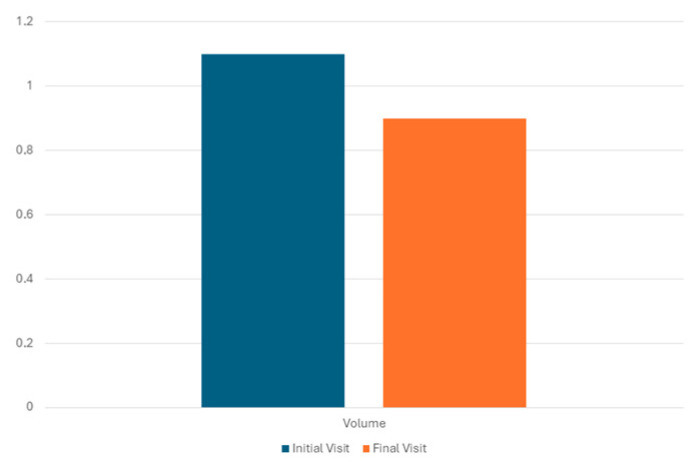
Histogram showing mean volume of lesion (expressed in mL) at initial and final visit.

**Figure 9:**
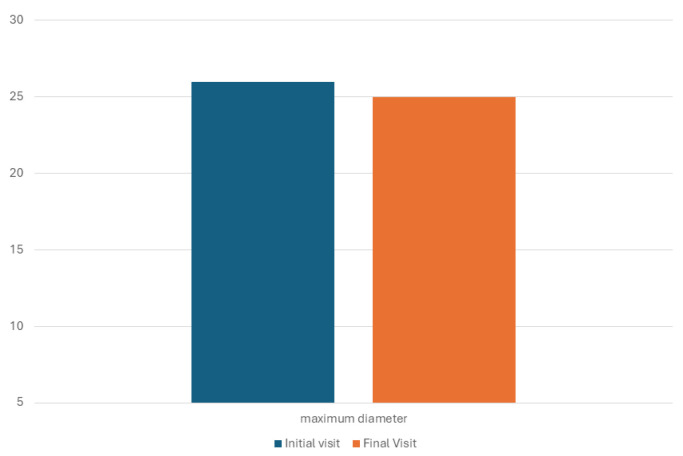
Histogram showing mean maximum diameter of lesion (expressed in mm) at initial and final visit.

Regarding the lesion size at the final visit, the median maximum diameter was 25 mm (interquartile range: 16, range: 8 to 80 mm), whereas the median lesion volume was 0.9 mL (interquartile range: 1.3, range: 0.05 to 37.0 mL). There was a significant reduction in the lesion volume (*p* = 0.017) (Figure 8). However, the maximum diameter did not change significantly (*p* = 0.779) (Figure 9).

**Figure 10:**
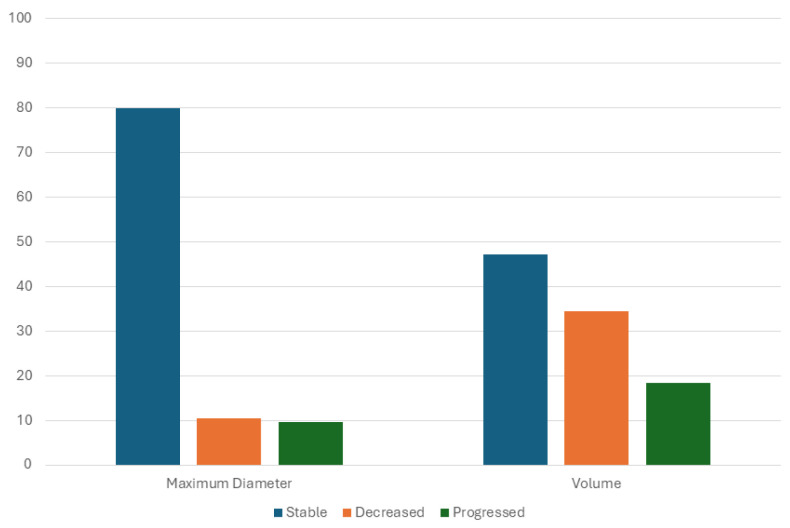
Distribution of stable, regressed, and progressed lesions according to maximum diameter of lesion and lesion volume.

Considering the variation in the lesion’s maximum diameter, 80.0% (n = 100) of the lesions remained stable, 10.4% (n = 13) decreased, and 9.6% (n = 9) progressed. Considering the variation in the lesion volume, 47.2% (n = 59) of the lesions remained stable, 34.4% (n = 43) decreased, and 18.4% (n = 23) progressed (Figure 10).

## 4. Discussion

In this study, we observed that continuous medical therapy with Dienogest significantly relieved patients’ painful symptoms, such as dysmenorrhea, dyspareunia, and dyschezia. We observed that dysmenorrhea, dyspareunia, and dyschezia improved in 85%, 66%, and 82% of women, respectively.

Regarding symptom relief, our findings agree with the data reported so far. In 2021, Vercellini et al. presented a narrative review about medical therapy in women with bowel endometriosis [8]. According to these authors, analyzing data from 10 studies comprising 588 women with proximal and/or distal sigmoid involvement, the probability of partial or complete relief was 100% for diarrhea and the passage of mucous, 98% for constipation, 90% for a feeling of incomplete evacuation and cyclic hematochezia, 82% for intestinal cramping, and 79% for abdominal bloating. In addition, dysmenorrhea subsided in 80% of the considered women, deep dyspareunia in 78%, and non-cyclic pelvic pain in 67%. Interestingly, the risk of bowel occlusion during medical therapy was less than 1%.

Furthermore, Ceccaroni et al. recently found that medical therapy in women with bowel endometriosis improved quality of life in a series of 580 women [20]. Therefore, it seems that medical therapy can be considered in women with non-occlusive symptoms and no intention of conceiving in the short term [8].

Regarding the main objective of our study, we observed that most lesions remained stable at long-term follow-up. However, we noted an interesting finding. We did not find significant changes in the lesion size when we considered the maximum diameter of the lesion. However, when considering the lesion’s volume, we observed that the median significantly decreased during follow-up. This could be explained by the fact that the volume better reflects the actual size of the lesion rather than a single diameter. In fact, we observed that 80% of lesions remained stable, 10% decreased, and 10% progressed when the maximum lesion size criterion was used to assess the lesion’s size. However, if the volume is considered, 34% of lesions decreased and 18% progressed. We believe that this finding is relevant, since these parameters cannot be used indistinctly to assess lesion evolution. In our opinion, the lesion’s volume is preferable as it can provide more reliable information about the actual lesion size.

To the best of our knowledge, few studies have assessed long-term size changes in recto-sigmoid endometriotic lesions.

Netter and co-workers retrospectively evaluated the natural progression of deep endometriosis nodules infiltrating the recto-sigmoid colon in women who did not undergo surgery, between two magnetic resonance imaging (MRI) scans separated by at least 12 months, in 43 women [21]. The mean follow-up duration was 38.3 months between the two MRI scans. Nodules were classified as progressive, regressive, or stable based on changes in length or thickness of ≥20%. Most patients underwent different types of medical treatment (oral contraceptives or GnRH analogs). Women who became pregnant were not excluded. No patient underwent surgery during follow-up. These authors observed that 28% of patients showed lesion progression, 60% remained stable, and 12% showed regression. The factor most associated with progression was the absence of amenorrhea: patients with a larger proportion of time in amenorrhea (induced by hormonal treatments, pregnancy, or breastfeeding) had a significantly lower risk of progression. The study concluded that continuous amenorrhea induction may have a protective effect against the progression of recto-sigmoid nodules, supporting the role of medical treatment as a conservative strategy in certain patients.

Egekvist and colleagues conducted a prospective study evaluating clinical and ultrasound outcomes in a series of 80 women with deep endometriosis of the recto-sigmoid colon, treated medically with combined oral contraceptives (COCs), oral progestins (OGs), or levonorgestrel-releasing intrauterine devices (LNG-IUDs) [22]. All women completed the 12-month follow-up. No association was observed between changes in nodule size and symptoms. These authors assessed the lesion volume using the VOCAL^TM^ method (GE Healthcare, Zipf, Austria) and the lesion length. Patients did not undergo bowel preparation before the ultrasound exam. They did not observe significant changes in lesion length. Egekvist et al. observed that, considering the lesion volume, 19% of women had lesion progression and 22.5% of patients had lesion regression.

Barra and colleagues reported a retrospective analysis including 83 women with deep endometriosis nodules in the recto-sigmoid region who were offered medical therapy with Dienogest 2 mg daily [23]. Apparently, bowel preparation before ultrasound examination was performed. The presence of recto-sigmoid endometriosis was confirmed by rectal water-contrast transvaginal ultrasonography. The largest diameter of the lesion and the lesion volume were used to assess lesion evolution. The lesion volume was estimated using the VOCAL^TM^ method. Only 34 women completed the 36-month follow-up. These authors observed that, after the end of the first year of treatment, a statistical decrease in the largest diameter and nodule volume was observed. However, after 24 and 36 months of treatment, the largest diameter and volume of endometriotic nodules remained stable. At the end of the study, in 52.9% of patients, there was a decrease in volume of at least 10%; in 35.3% of them, the lesion volume remained unchanged (+10%); and, in 11.8% of them, an increase in the lesion volume was observed in comparison to the baseline values.

Abrao and co-workers performed a retrospective study analyzing the clinical and ultrasound progression of recto-sigmoid endometriosis in 164 women awaiting surgery and treated medically (n = 90) (oral progestins, oral combined contraceptives, or GnRH analogs) or expectantly (n = 74) (no treatment) for at least 36 months (median follow-up was 47.6 months) [24]. All were evaluated by transvaginal ultrasound with bowel preparation. The nodule length and circumference were measured. No significant progression in lesion size was observed during follow-up. No differences were found between patients with and without hormonal treatment. However, postmenopausal women showed a significant reduction in nodule size.

Finally, Keckstein and colleagues reported a retrospective analysis comprising data from 38 women with recto-sigmoid endometriotic nodules managed conservatively [25]. The median follow-up period was 7.2 years. Several medical therapies were used, but not all patients had medical therapy. In fact, only 39% of women were still under medical therapy at the end of the study. The nodule length and thickness were measured, correlating them with age and the cumulative duration of hormonal therapy. The results showed that lesions tended to increase in size until the end of the fourth decade of life, stabilizing thereafter. In contrast, the duration of hormonal therapy had a significant regressive effect on the nodule size (both length and thickness). The statistical model confirmed that the longer the duration of hormonal treatment, the greater the likelihood of nodule regression.

Although there are clear differences regarding study design among these studies, we consider that our findings regarding lesion size changes agree with the data reported in the literature. Most studies observe no change in the maximum diameter or length of the lesion in women undergoing medical therapy. In contrast, when the lesion volume is considered, about 22–52% of the lesions regressed and about 11–19% of lesions progressed.

Certainly, there are limitations in interpreting these findings. First, only two studies used one single therapy (Dienogest). According to these studies (Barra’s study and the present one), in this therapy, most lesions remain stable or regress. However, most studies report data across several types of treatments. Therefore, it is difficult to ascertain whether there are different effects depending on the therapy used. In fact, we did not compare the effects of Dienogest on the lesion size with those of other medical therapies, or the effects of Dienogest on clinical symptoms as compared to surgery or other medical therapies, which could weaken the clinical applicability of our results.

Second, there are conflicting data regarding the reproducibility of ultrasound measurements. We found good reproducibility for lesion measurement and volume estimation. This is in agreement with data reported by Bean et al. [26]. Netter at al. also found high reproducibility using MRI [21]. However, Egekvist et al. reported moderate reproducibility for these measurements [27]. In fact, the use of bowel preparation differs among studies, so the external validation of the ultrasound scanning technique still needs to be performed.

Third, most studies are retrospective analyses and use different diagnostic approaches (for example, with and without bowel preparation prior to the ultrasound scan). Therefore, more and better-designed studies are needed.

Fourth, the lack of a control group hinders the generalizability of the results obtained. Furthermore, we had a significant proportion of cases lost to follow-up (13.8%). This potentially could bias the results.

Fifth, we did not perform sample size calculation. Thus, the *p*-values observed should be taken with some caution.

Sixth, we did not assess physical examination findings. However, we wished to focus on the clinical symptoms and ultrasound findings. In addition, it is already known that physical examination findings do not correlate with ultrasound findings [28]

Finally, we acknowledge that some women underwent a hysterectomy during follow-up. This could have biased the results regarding dysmenorrhea complaints.

It is interesting to note that medical therapy in young women with deep endometriosis and no desire for pregnancy in the short term might aid in fertility preservation, as recently suggested by La Marca et al. [29]. This idea is in line with the recently developed concept of ovariostasis [30]. Ovariostasis is defined as the reversible and temporary suspension of cyclic ovarian activity, which is medically achieved in women of reproductive age through the administration of exogenous drugs. Ovariostasis is characterized by anovulation and hypogonadotropinemia. Ovariostasis plays a preventive role in several gynecological conditions. One of them is endometriosis. Therefore, medical therapy for young women suffering from recto-sigmoid endometriosis, with no indication for surgery and no wish to become pregnant in the short term, could be an option for fertility preservation. In fact, we observed that the likelihood of deep endometriosis nodule volume reduction was higher in younger women.

On the other hand, we believe that future research should focus on randomized trials comparing medical treatment versus surgery based on the clinical profile, standardization regarding the clinically relevant threshold for progression (the extent of growth required to necessitate surgery), and the long-term impact on fertility and quality of life.

## 5. Conclusions

Despite the limitations observed, several conclusions could be drawn:

1. Medical therapy based on progestins, COCs, or LNG-IUDs is effective in controlling pain in women with deep recto-sigmoid endometriosis;
2. Nodule progression is uncommon in those of childbearing age if continuous amenorrhea is achieved;
3. Ultrasound monitoring can detect morphological changes and guide clinical decisions;
4. In patients without an immediate desire for pregnancy or signs of obstruction, medical treatment may be a safe alternative to surgery;
5. The duration of therapy and adherence (assessed by sustained amenorrhea) are key determinants of success;
6. Lesion regression appears more likely in younger women and those with prolonged use of hormone therapy.

## Figures and Tables

**Table 1:** Distribution of symptoms at initial visit.

Symptom	N	Percentage
None	15	12.0%
Dysmenorrhea only	5	4.0%
Dyspareunia only	12	9.6%
Dyschezia only	3	2.4%
Dysmenorrhea and dyspareunia; no dyschezia	16	12.8%
Dysmenorrhea and dyschezia; no dyspareunia	9	7.2%
Dyspareunia and dyschezia; no dysmenorrhea	13	10.4%
Dysmenorrhea, dyspareunia, and dyschezia	52	41.6%
Total	125	100%

**Table 2:** Severity of symptoms at initial visit.

	Absent	Mild	Moderate	Severe	Total
Dysmenorrhea	33 (28.7%)	3 (2.6%)	14 (12.2%)	65 (56.5%)	115 (100%) *
Dyspareunia	32 (25.6%)	9 (7.2%)	42 (33.6%)	42 (33.6%)	125 (100%)
Dyschezia	48 (38.4%)	12 (9.6%)	22 (17.6%)	43 (34.4%)	125 (100%)

* Ten women had a hysterectomy before entering the study.

**Table 3:** Severity of symptoms at final visit.

	Absent	Mild	Moderate	Severe	Total
Dysmenorrhea	95 (82.6%)	3 (2.6%)	12 (10.4%)	5 (4.4%)	115 (100%) *
Dyspareunia	65 (52.0%)	17 (13.6%)	27 (21.6%)	16 (12.8%)	125 (100%)
Dyschezia	97 (77.6%)	7 (5.6%)	15 (12.0%)	6 (4.8%)	125 (100%)

* Including those 8 women who underwent a hysterectomy during follow-up.

**Table 4:** Distribution of dysmenorrhea severity at initial and final visit.

		Final Visit				Total
		Absent	Mild	Moderate	Severe	
Initial Visit	Absent	33	0	0	0	33
	Mild	4	0	0	0	3
	Moderate	10	1	3	0	14
	Severe	49	2	7	5	65
Total		95	3	12	5	115

**Table 5:** Distribution of dyspareunia severity at initial and final visit.

		Final Visit				Total
		Absent	Mild	Moderate	Severe	
Initial Visit	Absent	27	0	4	1	32
	Mild	4	1	3	1	9
	Moderate	20	10	11	1	42
	Severe	14	6	9	13	42
Total		65	17	27	16	125

**Table 6:** Distribution of dyschezia severity at initial and final visit.

		Final Visit				Total
		Absent	Mild	Moderate	Severe	
Initial Visit	Absent	45	2	1	0	48
	Mild	10	1	0	1	12
	Moderate	15	2	4	1	22
	Severe	27	2	10	4	43
Total		97	7	15	6	125

**Table 7:** Measurements for interobserver analysis.

		Median	IQR	Range	*p*-Value
Maximum lesion diameter					
	Examiner A	22.0 mm	11	14–35 mm	0.118
	Examiner B	21.0 mm	11	14–34 mm	
Lesion volume					
	Examiner A	2.09 mL	5.3	0.72–10.72 mL	0.07
	Examiner B	2.08 mL	5.0	0.72–11.40 mL	

IQR: interquartile range.

**Table 8:** Maximum diameter and volume of lesion at initial visit according to severity of dysmenorrhea.

	Absent/Mild †	Moderate	Severe	*p*-Value
Maximum diameter (mm) *	21.00 (17.00)	32.50 (13.00)	26.00 (17.00)	0.058
Volume (mL) **	0.70 (1.65)	1.45 (3.00)	1.20 (1.15)	0.093

* Expressed as median, with interquartile range in parentheses. ** Expressed as median, with interquartile range in parentheses. † Mild cases analyzed jointly with absent cases due to the low number of mild cases (*n* = 3).

**Table 9:** Maximum diameter and volume of lesion at initial visit according to severity of dyspareunia.

	Absent	Mild	Moderate	Severe	*p*-Value
Maximum diameter (mm) *	23.00 (21.00)	30.00 (13.00)	27.00 (16.00)	25.50 (16.00)	0.859
Volume (mL) **	1.20 (2.23)	1.10 (0.55)	1.30 (1.25)	0.80 (1.15)	0.716

* Expressed as median, with interquartile range in parentheses. ** Expressed as median, with interquartile range in parentheses.

**Table 10:** Maximum diameter and volume of lesion at initial visit according to severity of dyschezia.

	Absent	Mild	Moderate	Severe	*p*-Value
Maximum diameter (mm) *	27.00 (17.00)	24.00 (13.00)	24.50 (17.00)	27.00 (17.00)	0.925
Volume (mL) **	0.90 (1.68)	1.15 (2.23)	1.25 (2.35)	1.10 (1.10)	0.793

* Expressed as median, with interquartile range in parentheses. ** Expressed as median, with interquartile range in parentheses.

## Data Availability

Data are available from the authors upon reasonable request.

## Abbreviations

The following abbreviations are used in this manuscript:

DE	Deep endometriosis
TVS	Transvaginal sonography
